# Walking for transportation in large Latin American cities: walking-only trips and total walking events and their sociodemographic correlates

**DOI:** 10.1080/01441647.2021.1966552

**Published:** 2021-08-14

**Authors:** Xavier Delclòs-Alió, Daniel A. Rodríguez, Catalina Medina, J. Jaime Miranda, Ione Avila-Palencia, Felipe Targa, Mika R. Moran, Olga Lucía Sarmiento, D. Alex Quistberg

**Affiliations:** a Institute of Urban and Regional Development, University of California, Berkeley, CA, USA; b Department of City and Regional Planning & Institute for Transportation Studies, University of California, Berkeley, CA, USA; c Center for Nutrition and Health Research, National Institute of Public Health, Cuernavaca, Mexico; d CRONICAS Center of Excellence in Chronic Diseases, Universidad Peruana Cayetano Heredia, Lima, Peru; e Urban Health Collaborative, Drexel University, Philadelphia, PA, USA; f World Bank, Washington DC, USA; g Department of Epidemiology, Universidad de los Andes, Bogota, Colombia.

**Keywords:** Mobility, pedestrian, physical activity, active transportation, travel surveys, urban areas

## Abstract

Walking for transportation is a common and accessible means of achieving recommended physical activity levels, while providing important social and environmental co-benefits. Even though walking in rapidly growing urban areas has become especially challenging given the increasing dependence on motorised transportation, walking remains a major mode of transportation in Latin American cities. In this paper we aimed to quantify self-reported walking for transportation in Mexico City, Bogota, Santiago de Chile, Sao Paulo, and Buenos Aires, by identifying both walking trips that are conducted entirely on foot and walking events involved in trips mainly conducted on other means of transportation (e.g. private vehicle, public transit) among individuals ≥5-years old. We show how walking-only trips account for approximately 30% trips in the analysed cities, and we evidence how the pedestrian dimension of mobility is largely underestimated if walking that is incidental to other transportation modes is not accounted for: when considering all walking events, we observed an increase between 73% and 217% in daily walking time. As a result, we estimated that between 19% and 25% of residents in these cities meet the WHO physical activity guidelines solely from walking for transportation. The results of the study also suggest that the promotion of public transportation in large Latin American cities can especially help certain population groups achieve the daily recommended levels of physical activity, while among low-income groups accessibility and safety seem to be the key challenges to be addressed.

## 1. Introduction

Physical inactivity is one of the lead causes for non-communicable diseases world-wide (Lee et al., 2012), and results in considerable economic costs for health care systems (Ding et al., 2016). Globally, 28% of adults aged 18+ is inactive or have sedentary lifestyles (WHO, 2016). This percentage is higher in some Latin American countries such as Brazil (47%), Colombia (44%) and Argentina (42%), and is over 80% among children (WHO, 2016). Daily physical activity (PA) among adults is associated with reductions in the risk of health problems including coronary heart disease, type 2 diabetes, certain types of cancer, and can also prevent mental health problems such as depression (Sun, Norman, & While, 2013). Among children, PA is essential to ensure growth and an adequate development (Hills, King, & Armstrong, 2007). Consequently, the World Health Organisation (WHO) recommends moderate-intensity PA for at least 150 min per week for adults and 300 min for children (WHO, 2010).

Among the different domains of PA (i.e. leisure and recreation, transportation, home related activities, and work), walking for transportation is a common and accessible means of contributing to reaching recommended PA levels (Spinney, Millward, & Scott, 2012). At the same time, walking has also gained relevance in the urban and transportation planning spheres, increasingly framed under the scope of environmental and social sustainability (Banister, 2008; Sallis, Frank, Saelens, & Kraft, 2004). On the one hand, active transportation modes such as walking or cycling are undoubtedly the most efficient means in terms of energy consumption, as well as the modes of transportation that are closer to being emission-free (Duffy & Crawford, 2013). On the other hand, walking in particular also has implications in terms of social equity. Walking can be regarded as the most democratic means of transportation, since it grants universal accessibility as almost everyone can be a pedestrian regardless of age, gender, ethnicity or socioeconomic status (Curtis & Scheurer, 2010; Hanson, 2010). In this sense, urban spaces that are conducive of pedestrian activity can help diminishing social inequalities as caused by unequal access to other transportation options (El-Geneidy & Levinson, 2011; Marquet & Miralles-Guasch, 2014). However, daily walking became challenging during the twentieth century, with urban areas rapidly growing as land uses were increasingly separated and mobility grew more dependent on motorised transportation. In Latin America, haphazard processes of urbanisation have resulted in large and dispersed cities, but land use mix and population densities have remained relatively high (Inostroza, Baur, & Csaplovics, 2013), thus walking is still very prevalent in the region (Ferrari et al., 2020; Herrmann-Lunecke, Mora, & Sagaris, 2020). In this sense, while most Latin American cities present relatively “sustainable” transportation systems in terms of modal shares of trips, this is generally not necessarily a consequence of better public and active transportation infrastructure, but rather as a consequence of lower motorisation rates compared to other geographic contexts (Tirachini, 2019).

Owing to the importance of walking for transportation, there is increased attention to understand current population levels of walking. This mirrors growth in studies exploring daily physical activity engagement among residents of Latin American cities (Salvo, Reis, Sarmiento, & Pratt, 2014, 2017; Werneck et al., 2019), and especially those looking at walking as a source of physical activity (de Sá et al., 2017; Ferrari et al., 2020). Previous studies about walking activity in Latin American has generally relied on measures such as daily or weekly walking time or the proportion of individuals achieving the recommended PA guidelines from walking, which are obtained from standardised physical activity surveys such as IPAQ (Florindo et al., 2019; Hallal et al., 2010). In our view, a deeper understanding of walking for transportation in the region is still needed, mainly in terms of why and how walking for transportation occurs. This means exploring walking trip frequencies, trip purposes, socioeconomic differences in walking and the relationship between walking and other transportation modes. In this same line, a recent review pointed out how studies exploring accessibility issues in Latin American cities should pay more attention to active modes in order to identify paths to improve the conditions in which these trips occur, and thus inform environmental and socially sustainable transport planning in developing countries (Vecchio, Tiznado-Aitken, & Hurtubia, 2020). This is particularly relevant for individuals in low income groups and also women, who not only are more likely to walk for transportation on a daily basis (Herrmann-Lunecke et al., 2020; Marquet, Bedoya, & Miralles-Guasch, 2017) but also have specific experiences of walking (Figueroa Martínez, Hodgson, Mullen, & Timms, 2019; Sagaris & Tiznado-Aitken, 2018).

Household travel surveys are a common source of data used not only in transportation studies, but also housing, environmental and health studies given their representativeness of the large population, their spatial resolution, as well as their rich account of personal, contextual and mobility characteristics. However, household travel surveys are generally considered to underestimate walking activity. A first reason for this underestimate is that some surveys are designed to collect data on longer trips or motorised trips only (Stopher, Xu, & Fitzgerald, 2007). Second, short walking trips (e.g. walking from home to store) are less likely to be reported, perhaps because they are less memorable, and thus more susceptible to recall bias (Saelens, Moudon, Kang, Hurvitz, & Zhou, 2014). Third, multimodal trips that include walking activity (e.g. walking to the subway, to transfer from one bus to another or from a station to the final destination) tend to be underreported in these surveys (Agrawal & Schimek, 2007). The latter might be a particularly significant source of concern, considering that previous studies have demonstrated an association between public transportation use and walking (Besser & Dannenberg, 2005; Karusisi, Thomas, Méline, Brondeel, & Chaix, 2014; Lemoine et al., 2016; Sallis et al., 2016). This may be especially relevant in Latin America, where transit use is considerably higher relative to other world regions (Salvo et al., 2014).

In this study we offer a detailed description of walking for transportation of individuals ≥5-years old in five large Latin American cities from different countries, using recent household travel surveys. We first provide a general picture of walking by analysing self-reported walking-only trips. Second, for a subset of cities with available data, we drill deeper to measure all walking activity related to transportation, including multimodal trips where walking was at least one of the modes used. Comparing walking-only trips to overall walking for transportation is helpful to understand how much walking is incidental to other modes of transportation vis-à-vis walking-only trips. Understanding how many walking events and how much time is spent daily on walking for transportation is critical for accurately quantifying the benefits and costs of transportation options, including those reliant on walking for access or egress. Thus, the contribution of this study is three-fold: first, we aim to quantify how much walking for transportation takes place in a sample of large Latin American cities; second, we aim to quantify how much of that walking is related to the use of other modes of transportation; third, we intend to highlight how much does walking for transportation contribute to achieving public health guidelines on physical activity. Furthermore, we examine differences across walking outcomes by a set of sociodemographic characteristics. This description is valuable in the context of some of the largest cities in the world, and in the most sedentary and most urbanised region of the world. More specifically, in line with similar efforts in other world regions (Agrawal & Schimek, 2007; Gascon et al., 2019), we aim to be useful both to serve as context for other studies examining walking for transportation in urban settings in Latin America, and to inform regional policies that aim at improving accessibility and at increasing physical activity by means of promotion daily walking for transportation.

## 2. Materials and methods

### 2.1. Data sources and sample

We examined walking for transportation in five of the largest cities in Latin America, which together account for approximately 65 million people. We selected the largest cities in Latin America with household travel surveys available after 2010. Selected cities and survey years are Mexico City 2017, Bogota 2019, Santiago de Chile 2012, Sao Paulo 2017, and Buenos Aires 2010. A summary of city characteristics and methodological details for each survey are available in Supplemental Table S1.

Considering different sampling frames across surveys, we analysed walking patterns for individuals that are 5 years old or older. Even though the mobility patterns of individuals younger than 18 years old, and especially children, are particularly dependent of that of their parents (Veitch et al., 2017), travel surveys used in this study recorded trips separately for each traveller within a household.

### 2.2. Definition of walking-only trips (WOT) and total walking events (TWE)

We focused on walking-only trips (WOT) and total walking events (TWE). WOT refer to trips that are conducted entirely on foot, from a given origin to a given destination. However, there are certain differences in the definition of walking trips in each survey (Supplemental Table S1). TWE are commonly known as segments or stages in household travel surveys and refer to all types of walking activity related to transportation, including when walking is used to access, transfer between, or egress from other modes of transportation. Therefore, WOT are a subset of TWE.

All five of the selected household travel surveys provided sufficient information to analyse WOT, which corresponds to the first stage of the analysis. By doing this, we aimed to provide a broader account of what WOT represent in cities from different Latin American countries. For the second stage of the analysis, which explores TWE and its relationship with WOT, we focused on the three cities with available data: Mexico City, Bogota, and Santiago de Chile. For the analysis of WOT the final unweighted sample consisted of 455,776 individuals, representative of a population of 66,221,908 residents in these cities. For the analysis of TWE, the final sample consisted of 308,134 individuals, representative of 34,820,736 inhabitants.

Furthermore, our analysis was focused on weekdays (Monday through Friday) due to differences in sample definitions across surveys, and differences in the travel behaviour from weekdays to weekends (Agarwal, 2004).

### 2.3. Indicators of walking for transportation

We used a set of indicators for WOT and TWE to describe walking for transportation. For WOT we used:

- WOT: Number of daily walking-only trips per person.
- %WOT: Percentage of all daily trips that are walking-only.
- TWOT: Daily time spent in walking-only trips per person.
- %GT: Percentage of population meeting the WHO physical activity guidelines from walking-only trips. The WHO recommends 150 min of weekly PA for adults and 300 min for minors. Considering the specific examples provided by WHO on how to achieve such guidelines (WHO, 2010) a threshold of 30 min of daily walking time is used for adults and 60 min is applied to minors.
- Trip purposes (percentage): Since each survey presents a specific definition of trip purposes, we harmonised the data across surveys (Supplementary Table S3). The resulting trip purposes were work/study, errands, recreational destinations, other and return home. Lastly, the share of trips labelled as “other”, those conducted to return home and those with an unknown answer were also calculated but are not shown in the tables.

For the analysis of TWE, we calculated the following indicators:

- TWE: Number of daily total walking events per person. TWE refers to all walking activity, including both WOT and walking activity that takes place before, during or after trips on other modes.
- %TWE: Percentage of all daily transportation events that are walking. Transportation events correspond to all trip stages, even if there are no walking events between stages conducted in other modes (e.g. stage conducted by bus and a different stage conducted by a different motorised means of transportation).
- %M: Percentage of total walking events that are part of a multimodal trip.
- TTWE: Daily time spent in total walking events per person.
- %GE: Percentage of population meeting the WHO physical activity guidelines considering all walking events.

### 2.3. Sociodemographic characteristics

We stratified the indicators for both WOT and TWE outcomes by sex, age, education level, socioeconomic status (SES), and car ownership at the household level. Sex, age, and car ownership were self-reported. Educational levels and SES were harmonised based on survey-specific definitions of each variable (Supplemental Tables S4 and S5). Education levels were then transformed to age-appropriate education level, matching each education level to its corresponding age group.

Sociodemographic characteristics of respondents are presented in Table 1. A slightly higher share of women (51-53%) than men was included in the sample. In terms of age, all cities presented a similar pattern, with similar shares of minors (<18 years old; 18-22%) and young adults (18-19 years old; 20-22%), a larger share of adults between 30 and 64 years old (45-51%) and a smaller share of older adults (65=< years old; 9-13%). Most the survey respondents reported an age-appropriate education level, but in Buenos Aires this proportion was lower (59%). In terms of SES, the population distribution is similar for Mexico City, Bogota, and Sao Paulo, for which most individuals were classified within the lowest SES group (41-56%). In Santiago de Chile, most of the sample was classified as high SES (43.4%), and for Buenos Aires most individuals fell in the middle SES group (40.4%). Lastly, most individuals in Mexico City, Bogota and Buenos Aires reported no car ownership at the household level (57-65%), while in Santiago de Chile and Sao Paulo most individuals reported car ownership (51-58%).

**Table 1.** Sample characteristics stratified by city.

	Mexico City 2017	Bogota 2019	Santiago de Chile 2012	Sao Paulo 2017	Buenos Aires 2010
**Sample Size N**	188,564	63,244	56,326	82,239	65,403
**Population N^a^**	19,690,219	8,939,564	6,190,953	19,356,755	12,044,417
**Total (%^a^)**	100	100	100	100	100
**Sex (%^a^)**					
Men	48.2	46.9	48.8	47.1	47.5
Women	51.8	53.1	51.2	52.9	52.5
**Age (%^a^)**					
<18	20.4	18.9	19.6	18.4	22.4
18–29	20.8	22.1	21.3	20.9	19.8
30–64	49.0	47.0	48.3	51.3	44.9
65=<	9.8	12.0	10.8	9.4	12.9
**Age-appropriate education level (%^a^)**					
No	16.3	19.5	13.2	29.3	41.2
Yes	83.5	80.5	85.9	70.7	58.5
**SES (%^a^)**					
Low	56.4	46.7	26.9	40.5	27.6
Middle	30.6	34.7	29.6	36.8	40.4
High	13.1	18.5	43.4	21.6	31.9
**Car ownership (%^a^)**					
No	57.0	64.8	48.8	42.2	59.6
Yes	43.0	35.2	51.2	57.8	40.4

^a^ Weighted using survey-specific population weights.

### 2.4. Analysis

We used estimated marginal means to describe each indicator for each demographic subgroup, while adjusting for all sociodemographic variables (gender, age, education level, SES and car ownership). Estimated marginal means, also known as “adjusted means”, are a practical way of conveying differences across groups for an outcome of interest. They are the predicted mean values for an outcome, calculated using the estimated model coefficients while varying a variable of interest (e.g. car ownership from 0 to 1) and holding all other covariates at fixed values. In other words, we report the mean predicted values for each outcome and independent variable of interest while adjusting for the effect of all other covariates simultaneously. We used negative binomial models to estimate the marginal mean count of WOT, the marginal proportion of %WOT and of WOT purposes, the marginal mean count of TWE, and the marginal proportion of %TWE and %M. Percentage variables (%WOT, WOT purposes, %TWE and %M) were modelled as counts with the corresponding total number of trips or events as an exposure (for example, for %WOT, we modelled the count of WOT with the total number of daily trips in all modes as the exposure, and for WOT purposes we modelled the count of WOT for each purpose and used the total number of WOT as the exposure). Predicted proportions were subsequently transformed to percentages and reported in the final tables. We used linear regression models to estimate marginal mean time of TWOT and TTWE. Lastly, considering that %GT and %GE took values of either 0 or 1 (whether the individual achieved WHO guidelines from WOT and for TWE, respectively), we used logistic regression models to estimate the average predicted probability of meeting standard (%GT and %GE). All models used robust standard errors.

All calculations were conducted using survey-specific population weights. Statistical analyses were performed using STATA 16 (StataCorp, 2016, College Station, Texas).

## 3. Results

### 3.1. Walking-only trips (WOT)

Estimated marginal means for WOT indicators, stratified by sociodemographic characteristics, are presented in Table 2. The estimated mean of WOT per person on weekdays was between 0.4 and 0.6, similar across the five cities. The estimated daily time spent in WOT (TWOT) ranges between 6.4 min (Sao Paulo) and 12.6 min (Bogota). WOT represented between 23.3% (Sao Paulo) and 37.7% (Bogota) of all trips performed during weekdays. Bogota had the highest proportion of population meeting WHO physical activity guidelines solely from WOT (12.6%), followed by Mexico City (10.4%), Santiago de Chile (7.7%), Buenos Aires (7.5%) and Sao Paulo (7.1%).

**Table 2.** Estimated marginal means of individual walking-only trip indicators stratified by city and adjusted by sociodemographic variables in the analysed cities.

	Daily walking-only trips per capita (WOT)					% of all trips that are walking-only (%WOT)					Daily time spent in walking-only trips (TWOT)					% of population meeting WHO PA guidelines from walking-only trips on weekdays (%GT)				
	MX17	BO19	SC12	SP17	BA10	MX17	BO19	SC12	SP17	BA10	MX17	BO19	SC12	SP17	BA10	MX17	BO19	SC12	SP17	BA10
**Total**	0.6	0.6	0.5	0.5	0.4	32.1	37.7	28.6	23.3	27.7	9.0	12.6	7.0	6.4	6.9	10.4	12.6	7.7	7.1	7.5
**Sex**																				
Men	0.4	0.4	0.4	0.5	0.3	22.7	30.4	23.3	22.4	22.0	6.6	9.9	6.1	6.2	5.6	6.1	9.5	6.0	6.2	5.3
Women	0.8	0.7	0.6	0.5	0.5	41.2	44.2	33.6	24.1	33.6	11.2	15.0	7.8	6.6	8.0	14.4	15.4	9.3	7.8	9.6
**Age**																				
<18	1.0	0.9	0.6	1.0	0.8	57.8	59.2	41.9	47.4	51.0	14.6	17.9	10.2	13.0	11.4	4.2	8.6	3.8	3.7	3.2
18–29	0.4	0.4	0.4	0.5	0.3	21.5	27.0	20.4	20.2	19.6	6.6	9.5	5.1	6.3	5.7	10.5	11.5	7.3	9.0	9.0
30–64	0.5	0.5	0.5	0.5	0.3	25.0	31.3	24.7	20.3	18.4	8.0	11.7	6.3	5.6	5.6	12.6	13.5	8.2	8.1	8.8
65=<	0.5	0.6	0.6	0.2	0.3	37.4	45.3	39.6	12.8	30.4	7.6	14.0	8.1	2.6	5.1	12.4	17.0	12.0	4.6	8.4
**Age-appropriate education level**																				
No	0.6	0.6	0.6	0.5	0.4	40.2	46.1	38.7	22.8	28.1	10.2	15.5	7.7	6.4	6.5	16.3	17.2	10.9	9.4	9.2
Yes	0.6	0.6	0.5	0.5	0.5	30.8	36.0	27.0	23.5	27.5	8.8	11.9	6.8	6.4	7.2	9.3	11.5	7.1	6.3	6.2
**SES**																				
Low	0.7	0.7	0.6	0.6	0.6	36.7	46.2	37.8	30.1	40.3	10.5	15.9	8.6	8.9	9.3	11.9	15.3	9.3	9.6	8.8
Middle	0.5	0.5	0.5	0.5	0.4	28.5	32.3	28.5	21.9	25.7	7.6	11.0	6.8	5.8	6.3	9.2	11.1	7.5	6.3	7.0
High	0.3	0.4	0.4	0.4	0.3	17.5	27.2	20.7	17.5	19.3	4.8	8.3	5.6	4.3	5.2	5.9	9.4	6.5	5.2	7.0
**Car ownership**																				
No	0.7	0.7	0.6	0.7	0.5	39.1	44.3	33.8	30.2	34.0	11.1	15.2	7.9	9.1	7.9	12.8	14.8	9.0	10.2	8.6
Yes	0.4	0.4	0.4	0.5	0.3	22.6	26.7	21.9	19.2	19.5	6.2	8.4	5.8	4.8	5.3	7.2	9.1	5.9	5.2	5.8

All figures are for weekdays. *P*-values of differences in the estimated values can be found in Supplemental Table S6.

After adjusting for all other individual and household characteristics, we observed significant differences in WOT across sociodemographic groups. Women consistently walked more than men across all cities (WOT per capita, %WOT and TWOT). In addition, women more frequently achieved the WHO PA guidelines only from WOT compared to men (7.8-15.4% vs. 5.3-9.5%).

Individuals aged <18 years old performed a higher amount of WOT than their adult counterparts. In addition, the percentage of WOT was higher among this group (41.9-59.2%). Furthermore, daily TWOT values went from 10 to 18 min among this group. However, only 3% to 9% of minors in the sample achieved the 60 min. recommendation of PA from WOT. In Bogota and Santiago de Chile, a higher number of older adults (65=< years old) more frequently achieved the recommended 30 min. of daily PA from WOT (17% and 12% respectively), while in Mexico it was the case for 30–49 year-old adults, and in Sao Paulo and Buenos Aires it was young adults (18-29 years old) who achieved such recommendations from walking-only trips in a higher proportion.

The relationships between estimated WOT indicators and education level and SES were generally consistent across all five cities. Individuals that did not report age-appropriate education levels and those with lowest SES had higher WOT and %WOT compared to all others. Average daily time spent on WOTs also decreased with education and SES, except in Sao Paulo and Buenos Aires in terms of age-appropriate education level. A higher percentage of individuals classified within the lowest SES and without age-appropriate education level achieved WHO PA guidelines only from WOT. In terms of car ownership, results are similarly consistent: individuals without a car in the household had higher WOT, %WOT, TWOT and %GT.

The main estimated purposes of trips for WOT were either to go to work or study locations or to run errands. Together these purposes account for between 45% and 50% of all WOT (Table 3). In Mexico City, Sao Paulo and Buenos Aires walk to work or study was the main purpose of WOT (26.4%-36.5%), while in Bogota and Santiago de Chile the main purpose of WOT was for errands (25-26%). Finally, between 3% and 9% of WOT were performed to access recreational destinations. The remainder proportions (44-49%) correspond to trips for other purposes and trips to return home, which are not displayed in the table.

**Table 3.** Estimated marginal means of individual walking-only trip purposes stratified by city and adjusted by socio-demographic variables in the analysed cities.

	% Work or study					% Errands					% Recreational destinations				
	MX17	BO19	SC12	SP17	BA10	MX17	BO19	SC12	SP17	BA10	MX17	BO19	SC12	SP17	BA10
**Total**	26.4	19.8	19.4	36.5	29.2	21.9	24.8	25.9	13.1	17.4	2.9	8.9	7.0	8.1	4.2
**Sex**															
Men	40.4	26.2	27.6	41.6	37.8	9.5	18.3	17.5	8.1	9.8	3.3	9.7	9.3	10.3	5.0
Women	18.1	15.4	14.1	31.8	23.0	29.3	29.2	31.3	17.7	22.9	2.7	8.5	5.6	6.1	3.7
**Age**															
<18	46.9	41.5	35.9	47.2	45.8	2.9	5.5	6.6	3.5	2.1	1.5	4.9	6.5	1.2	2.7
18–29	19.0	15.7	19.4	35.3	21.1	27.4	27.9	25.4	14.7	22.7	4.0	10.9	8.9	9.7	6.4
30–64	13.8	10.7	14.2	33.1	16.8	34.2	34.8	33.9	16.4	30.5	2.9	8.7	6.7	11.1	4.1
65=<	7.1	3.4	3.0	16.3	3.6	38.5	34.3	40.6	28.6	38.6	7.5	16.4	6.9	13.9	8.7
**Age-appropriate education level**															
No	12.1	8.4	7.3	28.1	18.7	35.6	34.1	37.9	20.8	26.9	3.8	10.3	4.9	3.9	4.9
Yes	29.3	22.6	21.9	38.8	35.3	19.1	22.5	23.4	11.0	11.9	2.8	8.6	7.5	9.2	3.8
**SES**															
Low	27.2	21.1	15.6	34.4	32.0	21.5	24.6	28.3	15.3	15.1	2.4	7.1	6.3	2.8	2.9
Middle	24.8	18.9	20.5	37.2	27.9	23.0	25.3	24.6	12.9	18.0	3.6	9.8	6.3	6.1	4.6
High	24.3	16.7	23.9	39.0	26.9	21.9	24.9	23.9	10.1	20.0	5.4	13.7	9.0	17.4	5.7
**Car ownership**															
No	27.4	20.8	18.4	34.8	29.5	21.4	24.4	27.0	15.2	17.6	2.5	8.1	5.8	4.6	4.1
Yes	24.0	17.2	21.3	38.0	28.8	23.2	26.1	24.0	11.3	16.8	3.8	11.1	9.3	11.0	4.5

All figures are for weekdays. Trips conducted to return home and trips characterised as “others” are considered in the calculations but not displayed on the table. *P*-values of differences in the estimated values can be found in Supplemental Table S7.

There are considerable differences in the main estimated walking trip purposes across sociodemographic characteristics. Relative to women, men consistently have higher shares of WOT for work or study, lower shares for errands, and higher shares of trips to recreational destinations. In terms of age, as traveller age increases the share of WOT for work or study decreases, while for errands it increases. For trips to recreational destinations, with the only exception of Santiago, older adults (65=< years old) generally have the highest share of WOT for this purpose. In terms of education, the distribution of WOT purposes are generally similar: individuals with age-appropriate education level showed higher shares of WOT for work or study, lower for errands and higher for WOT to recreational destinations. Conversely, in terms of SES, we observed how in Mexico, Bogota and Buenos Aires low SES individuals presented higher shares of WOT for work or study, while the opposite was observed in Santiago and Sao Paulo. However, it must be considered that in many cases differences in WOT purposes were not statistically significant for these two variables, especially in terms of age-appropriate education level (Supplemental Table S7).

Lastly, the relationship between car ownership in the household and WOT purposes also depends on the city. In general, car ownership was associated with lower shares of WOT conducted for work or study, except for Sao Paulo. In terms of errands, in Mexico City and Bogota the share of WOT for errands was smaller among those without a car in the household, while in the remaining three cities in the sample this association was the opposite. Across all five cities, however, individuals with car ownership at the household level consistently conducted a higher proportion of WOT to recreational destinations.

### 3.2. Total walking events (TWE)

When considering all walking events for Mexico City, Bogota, and Santiago de Chile, we observed a significant increase in the relevance of walking for transportation across all estimated indicators (Table 4). There were between 1.3 and 2 TWE on a weekday per person. TWE represented 47-67% of all transportation events. Of all TWE, between 54% and 68% were part of a trips conducted mainly in other modes (%M), and therefore between 32-46% of TWE were walking-only trips (WOT). Thus, approximately 60% of walking events in the analysed cities are conducted to either access, transfer between or egress from other transportation modes. On average, daily walking time spent in TWE (TTWE) was between 15.6 and 22.5 min per capita. In consequence, between 19% (Mexico City) and 25% (Bogota) of the population in these cities meet the WHO PA recommendations solely from walking for transportation when total walking events are considered.

**Table 4.** Estimated marginal means of individual walking event indicators stratified by city and adjusted by socio-demographic variables in the analysed cities.

	Daily total walking events per capita (TWE)			% of all events that are walking (%TWE)			% of total walking events that are part of a multimodal trip (%M)			Daily time spent in total walking events (TTWE)			% of population meeting WHO PA guidelines from total walking events on weekdays (%GE)		
	MX17	BO19	SC12	MX17	BO19	SC12	MX17	BO19	SC12	MX17	BO19	SC12	MX17	BO19	SC12
**Total**	1.3	2.0	1.9	46.5	66.5	55.2	53.8	63.8	67.5	15.6	22.5	22.1	19.0	25.0	22.9
**Sex**															
Men	1.2	1.8	1.8	40.2	62.1	50.4	62.3	68.5	70.4	13.8	19.3	20.9	15.7	20.9	20.9
Women	1.4	2.1	2.0	52.8	70.4	59.6	46.8	60.0	65.1	17.2	25.3	23.2	22.1	28.6	24.6
**Age**															
<18	1.4	1.7	1.4	63.6	75.2	58.2	28.5	40.8	47.4	18.0	22.7	19.3	4.7	10.4	6.3
18–29	1.5	2.1	2.4	42.4	62.3	56.5	70.7	75.4	80.2	16.0	21.3	25.0	24.3	27.5	29.4
30–64	1.3	2.1	2.0	40.8	63.5	51.8	60.3	70.4	70.9	15.2	22.8	22.8	23.0	28.4	26.1
65=<	0.9	1.7	1.6	48.5	70.6	62.2	45.4	57.3	58.2	11.5	22.9	19.2	18.0	28.6	24.0
**Age-appropriate education level**															
No	1.2	1.7	1.9	53.2	70.4	66.2	46.9	56.5	63.1	15.8	25.1	21.9	24.3	29.8	27.8
Yes	1.4	2.0	1.9	45.5	65.7	53.4	55.0	65.4	68.3	15.6	21.8	22.2	17.9	23.8	21.9
**SES**															
Low	1.4	2.0	2.0	50.4	70.9	63.8	50.7	56.6	62.8	17.2	26.4	23.8	20.5	28.7	23.7
Middle	1.4	2.0	2.0	45.5	64.2	56.3	58.9	68.9	68.3	14.8	20.8	21.7	18.8	23.5	22.9
High	0.8	1.8	1.8	29.4	60.2	46.4	59.1	72.9	71.5	9.4	16.8	20.9	11.7	19.4	22.0
**Car ownership**															
No	1.6	2.0	2.2	55.0	70.2	64.0	53.0	58.5	68.1	18.9	25.6	25.1	23.1	28.2	26.7
Yes	1.0	1.9	1.6	35.1	60.3	43.6	55.4	73.4	66.3	11.1	17.3	17.9	13.4	19.6	17.5

All figures are for weekdays. *P*-values of differences in the estimated values can be found in Supplemental Table S8.

Consistent with WOT, women had higher rates of walking for transportation (higher TWE, %TWE and TTWE) and therefore they met the WHO PA guidelines from all TWE more frequently than men. However, men’s walking events were more incidental to other transportation modes compared to women’s (%M).

While walking shares from TWE (%TWE) were still generally higher among minors, adults between 18 and 29 years old had the highest rates of TWE (1.5-2.4). This might be related to the significantly larger share of TWE that were part of multimodal trips among this group (%M between 70.7%-80.2%). Individuals without age-appropriate education level had lower TWE but higher %TWE, TTWE and more frequently achieved the WHO guidelines from TWE. More consistently, those within the lowest SES and those without access to a car had higher TWE, %TWE, TTWE and %GE. Individuals without age-appropriate education level, those in the lowest SES group and those without access to a car generally had the smallest shares of TWE that were part of multimodal trips (%M), suggesting that when low-income individuals walk, walking is more frequently their main mode of transportation.

### 3.3. Difference between walking-only trips (WOT) and walking events (TWE)

Walking for transportation was significantly underestimated when only WOT was considered (Table 5). Walking for transportation more than doubled in these three cities when all events were considered (the difference between estimated WOT and TWE ranged from 0.7–1.4). Accordingly, the proportion of all daily trips that were walking increased between 14.5 and 28.8 percent points when TWE were analysed. All these added walking events tend to be short trips. Thus, consideration of TWE resulted in an average increase of between 6.6 and 15.1 min per capita. This translated into increases in walking time for transport of 73% in Mexico City, 78% in Bogota, and 217% in Santiago when all walking was considered. In consequence, the share of population meeting the daily WHO PA recommendations from walking for transportation increased between 8.6 and 15.1 percent points if TWE were considered, reaching 19% of the population in Mexico City, 23% in Santiago, and 25% in Bogota (as shown previously in Table 4).

**Table 5.** Difference between estimated marginal means of total individual walking event and walking-only trip indicators, stratified by city in the analysed cities.

	Difference between number of total walking events and walking-only trips (TWE-WOT)			Difference between walking share considering TWE and WOT (%TWE-%WOT)			Difference between daily walking time considering TTWE and TWOT (TTWE-TWOT)			Difference in % of population meeting WHO PA guidelines on weekdays from TWE and WOT (%GE-%GT)		
	MX17	BO19	SC12	MX17	BO19	SC12	MX17	BO19	SC12	MX17	BO19	SC12
**Total**	0.7	1.4	1.4	14.5	28.8	26.5	6.6	9.8	15.1	8.6	12.4	15.1
**Sex**												
Men	0.8	1.4	1.4	17.5	31.7	27.1	7.2	9.4	14.8	9.6	11.4	14.9
Women	0.7	1.4	1.4	11.5	26.2	26.0	6.0	10.3	15.4	7.6	13.2	15.3
**Age**												
<18	0.4	0.8	0.8	5.9	16.1	16.3	3.5	4.9	9.1	0.5	1.8	2.5
18–29	1.1	1.7	2.0	20.9	35.3	36.1	9.4	11.8	19.9	13.7	16.0	22.1
30–64	0.8	1.6	1.5	15.8	32.3	27.1	7.2	11.1	16.5	10.3	14.9	17.8
65=<	0.4	1.1	1.0	11.1	25.4	22.7	4.0	8.9	11.0	5.6	11.6	12.0
**Age-appropriate education level**												
No	0.6	1.1	1.3	13.0	24.3	27.6	5.6	9.6	14.1	8.0	12.7	16.9
Yes	0.8	1.5	1.4	14.7	29.7	26.4	6.8	9.9	15.3	8.7	12.3	14.8
**SES**												
Low	0.7	1.3	1.4	13.7	24.6	26.1	6.7	10.5	15.2	8.6	13.4	14.4
Middle	0.9	1.5	1.5	17.0	31.9	27.8	7.2	9.8	14.9	9.6	12.4	15.5
High	0.5	1.4	1.4	11.9	33.0	25.7	4.6	8.6	15.4	5.8	10.0	15.5
**Car ownership**												
No	0.9	1.3	1.6	15.9	25.9	30.2	7.8	10.4	17.3	10.3	13.5	17.7
Yes	0.6	1.5	1.1	12.5	33.6	21.7	4.9	8.9	12.1	6.2	10.5	11.6

The difference between WOT and TWE, however, is not the same depending on sociodemographic characteristics of the sample. The most remarkable and consistent difference can be found in relation to age. Working-age adults (18-64), and especially younger adults (18-29), were particularly sensitive to the underestimation of walking activity if only WOT were considered, which is related to their higher shares of TWE that were part of multimodal trips (%M in Table 4). Similarly, daily walking time and, consequently, the percentage of population achieving the WHO guidelines, was especially underestimated for individuals without household car. For those with age-appropriate educational levels, the underestimation of walking events and time was also more underestimated, but this did not necessarily translate in a higher underestimation of the probability of achieving WHO guidelines. In relation to SES, we observed that even though it was individuals in higher SES groups who showed higher shares of walking that was incidental to other modes (%M in Table 4), the underestimation of overall walking time (and the share of individuals achieving the PA guidelines from walking for transport) was not as straightforward.

## 4. Discussion

In this study we provided a novel and detailed description of walking for transportation in large Latin American cities. To do so, we have relied on representative household travel surveys, which are based on large sample sizes. Contrasting the various ways in which walking can be measured, the purposes, and the implications for walking time and physical activity guidelines by socio-economic characteristics are distinctive contributions of the work presented.

When considering walking-only trips, we found that they are about one third of all daily trips. This figure is higher than in other parts of the world and lower than others. For instance, the share of walking trips in U.S. cities over three million inhabitants is close to 14%. In contrast, the share of WOT can be close to 50% in European cities like Barcelona (Delclòs-Alió & Miralles-Guasch, 2019) and up to 60% in African cities like Dakar or Douala (UITP International Association of Public Transport, 2011). Clearly these findings indicate differences in the spatial structure of cities as well as socio-economic conditions of the population. Latin American cities are among the densest in the world and motorisation still remains fairly low (OICA, 2015). This means that overall walking, and incidental walking particularly, are likely to contribute significantly to mobility. Similarly, as income rises trip-making and motorised trip-making also increases (Ksenofontov & Milyakin, 2018).

When total walking events were considered, the importance of walking for transportation more than doubled on average. Since the flexibility of car use mostly allows for door-to-door trips involving little walking, the difference observed is likely to be reflecting an underestimation of walking activity that happens as a way to access, transfer, and egress from mass transportation modes. Indeed, around 40% of daily travel in large Latin American cities is conducted on public transportation (Ferrari et al., 2020). The implications of these results are two-fold. First, from the perspective of transportation planning, the underestimation of walking when only WOT are considered underscores the importance of explicitly collecting detailed walking data when conducting household travel surveys. The fact that we were unable to identify walking events in two of the five surveys highlights the importance of these findings. As shown, such detailed data enables a deeper understanding of what is involved in trips made on other modes, allowing planners to develop a more accurate account of pedestrianism in these trips. This could help, for example, to consider how accesses and exits are designed in relation to their surrounding environments and how transfers could be made easier and more comfortable. In turn, properly accounting for the pedestrian dimension of transit ridership also informs policies that aim at increasing transit ridership by making walking more convenient, safer, and more attractive for transit stations' access, transfer, or egress.

Second, considering walking as often incidental to other travel modes is critical to understand the mechanisms through which the transportation domain contributes to physical activity. The results showed how the estimated time spent walking for transportation in our sample was between 15.6 (in Mexico City) and 22.5 (in Bogotá) minutes per day (see TTWE in Table 4). These figures are slightly lower compared to what Ferrari et al. (2020) found, which can be explained by differences in the data collection instrument and the fact that our study is focused on five of the largest Latin American cities. Considering that the underestimation of walking for transportation is likely to be related to transit use, the results of the study suggest a clear potential to reduce gaps in the achievement of physical activity recommendations by promoting the use of public transportation (MacDonald, Stokes, Cohen, Kofner, & Ridgeway, 2010; Miller et al., 2015).

Improving our ability to understand and monitor how walking for transportation contributes to public health is also important to bridge the transportation and health disciplines. Recent efforts describing how to connect the two fields identified transportation and health surveys as productive venues for joint collaboration (Medicine, 2019). Specifically, comprehensive inclusion of walking enables analyses that highlight the contributions of walking to physical activity, while allowing to account for the health benefits of walking and other modes related to walking, like public transportation use.

We found significant variations in walking for transportation across sociodemographic groups with important implications for transportation equity. First, in line with previous studies focused on urban areas (Ferrari et al., 2020; Maciejewska, McLafferty, & Preston, 2019), walking transportation was much more common among women than men. This could be explained by the fact that men still dominate car access at the household level (Gil Solá, 2016) and that women might shift away from public transportation due to cost and safety considerations (Orozco-Fontalvo, Soto, Arévalo, & Oviedo-Trespalacios, 2019). Accordingly, trip purposes related to walking for transportation are also different. For men, WOT were more frequently work-related than for women, and their share of walking activity that is incidental to other forms of transportation is also larger. Women, on the other hand, had larger shares of walking trips for running errands, which is consistent with the gendered assignment of household roles (Maciejewska, Marquet, & Miralles-Guasch, 2019), and is a pattern likely observed also across other modes of transportation. The finding that women walked proportionally less than men to recreational destinations is also noteworthy. This inequality could be again explained by the temporal constraints resulting from an uneven allocation of household responsibilities (Delclòs-Alió & Miralles-Guasch, 2018) and also possibly due to concerns related to safety from crime (Van Dyck et al., 2013). This result would also be consistent with a recent large-scale study using novel technologies that showed that men took more overall daily steps when not only walking for transportation was considered (Althoff et al., 2017), suggesting that some men have large share of walking for work or for recreation.

Individuals belonging to lower SES groups walked for transportation considerably more than others, which is also in line with previous studies (Brondeel, Pannier, & Chaix, 2016; Ferrari et al., 2020). This result may have positive health implications due to the higher ability of low SES individuals to meet daily PA levels from walking as a mode of transportation. However, these differences could also be reflecting deep inequalities in terms of spatial accessibility and thus the consideration of low SES individuals as “captive walkers” (Cervero, 2013). Our data supports this view in two ways: first, by showing that low SES travellers reported more walking-only trips than individuals in higher SES; second, by showing that when including all walking events, low and middle SES travellers still presented higher levels of overall walking time, even if incidental walking events among this group were significantly less common than among individuals in higher SES. These findings suggest that different socioeconomic groups require targeted approaches in terms of their relationship with walking for transportation. In this line, accessibility seems to be the key challenge to be addressed among lower SES groups, and thus efforts should be directed, for instance, at increasing spatial access to public transportation that would result in shorter walking trips to stops, while at the same time investing in walkable neighbourhoods that allow for shorter but more frequent walking trips in order to ensure the benefits of walking as a source of PA (Marquet, Hipp, & Miralles-Guasch, 2017).

Our findings also suggested that walking to recreational destinations appears to be a luxury and choice activity, considering it increased consistently with SES. This is in line with findings from previous studies, both in Latin America (Gomes et al., 2011) and in other urban contexts (Agrawal & Schimek, 2007; Yang, Diez Roux, Auchincloss, Rodriguez, & Brown, 2012). One possible explanation could be that individuals within lower SES groups have less economic resources and less time to allocate for recreational trips in general, and at the same time may be a result of an unequal distribution of recreational destinations, with high income individuals being more likely to have higher access to such destinations in a walkable distance from home. Another explanation is that higher shares of walking for transportation for work or study purposes may disincentivize walking also to recreational destinations.

Finally, age was associated with walking differently for WOT and TWE. First, we observed a higher relevance of WOT for individuals under 18 years old and, second, TWE was especially higher among young adults (18-29). The reason for the former is that walking-only trips are much common among children and teenagers compared to other and mostly refer to on active commuting to school (Sarmiento et al., 2015). It is hence particularly important to take into account the characteristics of the environments in which these trips take place (Aguilar-Farias et al., 2018), both in terms of perceived safety (Moran, Rodríguez, & Corburn, 2018) as well as the potential exposure of children and teenagers to transport-related pollutants and traffic collisions (Alvarez-Pedrerol et al., 2017; Rothman, Buliung, Macarthur, To, & Howard, 2014). When TWE were considered, middle-aged adults and especially younger adults significantly increased their walking times. The fact that this demographic group was the most affected by the underestimation of walking when only WOT are considered can again be largely explained by the fact that transit use is generally higher among this group (Delclòs-Alió & Miralles-Guasch, 2019). This suggests a clear potential of transit use as a form to engage in PA among working adults, especially considering that temporal constraints of increasingly longer workdays and longer commutes may limit the amount of time available to conduct other activities (Halonen et al., 2020).

This study has limitations to acknowledge that could inform the design of future studies. First, we were only able to quantify TWE for a sub-set of cities. Second, the walking behaviours measured were based on self-reports. These self-reports are known to be generally affected by recall biases, but are also and specifically likely to underestimate walking trips (Zmud & Wolf, 2003). As a response, household travel surveys have increasingly incorporated objective measures of travel patterns to complement self-reported accounts of daily travel activity, mainly based on high-resolution technologies such as GPS devices and, more recently, smartphone apps (Delclòs-Alió, Marquet, & Miralles-Guasch, 2017; Shen & Stopher, 2014). Third, some of the surveys used in the study explicitly omit short walking trips (Supplemental Table S1). Thus, not only this might partially limit comparability across cities, but it is also likely that actual levels of walking may be even higher than what we have described here. The clearest example is the case of Sao Paulo, where the significantly higher share of WOT conducted for work or study compared to other cities (and the consequent lower share of errands) might be partially explained by the omission of personal walking trips of under 500 meters. Fourth, we harmonised definitions of trip purposes, education levels and socioeconomic status based on differing definitions provided in each survey, which could also be considered as a potential source of bias (Supplemental Tables S3-S5). The categorisation of trip purposes in traditional classes such as work/study, errands and recreational destinations also has limitations. On the one hand, these categories are designed under the traditional consideration of work as “paid” work, which underestimates the relevance of tasks related to care, generally performed by women (Herrmann-Lunecke et al., 2020; Sagaris & Tiznado-Aitken, 2018). In the same line, this trip purpose categorisation generally does not allow to correctly capture all trips that satisfy more than one purpose (e.g. dropping off children at school on the way to work), which are also generally conducted by women (Scheiner & Holz-Rau, 2017). Fifth, although we used the most recent available surveys in each city, the years in which they were conducted differ. In addition, differences in the sample frame, sample selection and survey implementation are all likely to influence the results. Finally, we estimated a set of walking for transportation indicators considering only a selection of key sociodemographic characteristics. Other variables that could be especially relevant in the Latin American context, for example the built and social environments (Marquet, Bedoya, et al., 2017), should be incorporated in future studies aiming to compare WOT and TWE.

Our focus was on some of the largest cities in Latin America. However, it would be interesting for future studies to explore differences between walking-only trips and total walking events in small and medium-sized cities, where the provision of mass transportation options may be more limited (Allport & Thomson, 1990) and where research on walking and equity is still needed (Vecchio et al., 2020). Similarly, given prevailing differences in walking relative to respondent sex and SES, future studies should explore further how built and social environment variables may interact with these personal characteristics for both walking-only trips and all walking events. In this sense, it is particularly relevant to understand spatial differences in walking behaviour (both WOT and TWE) at a micro-scale (Vich, Marquet, & Miralles-Guasch, 2019), and in growingly car-dominated urban environments, something that has been made especially evident in the context of the COVID-19 global pandemic and the emerge of many initiatives reclaiming public space from motor vehicles to pedestrians (Honey-Rosés et al., 2020). Special attention should be paid to the walking experiences of women and those in lower SES groups (Figueroa Martínez et al., 2019; Vecchio et al., 2020), considering that despite the evident health benefits of longer daily walking times in terms of physical activity, the conditions in which walking occurs may at the same time have negative physical or mental health repercussions, for instance in terms of air quality or personal safety.

## 5. Conclusion

Walking is a significant part of everyday mobility in large Latin American cities. However, walking for transportation is not limited to walking-only trips. Instead, a significant part of walking takes place as part of trips conducted on other transportation modes. This study has shown the usefulness of comprehensively measuring walking for transportation in household travel surveys and its relevance for meeting public health guidelines on physical activity. When all walking is considered, including incidental walking in addition to walking-only trips, walking time more than doubles. As a result, we estimated that between 19% and 25% of residents in the analysed cities meet WHO physical activity guidelines solely from walking as a form of transportation. This is particularly important for low-income groups who spend more time walking, travel less via other modes, and meet PA guidelines from transportation more frequently than other groups. Future research should examine the city and neighbourhood contexts in which such walking occurs, and does not occur, to consider ways to supporting walking and making it safer. Given the importance of public transportation for walking, the findings from our work also suggest that promoting transit in large Latin American cities not only has the potential to address social and environmental challenges that the currently car-dominated transportation system poses, but also it can help certain segments of the population to achieve the daily recommended levels of physical. These questions are critically important as Latin America is the most urbanised and most sedentary region in the world, and thus highlighting the importance of walking for transportation has the considerable potential to alter public policy and improve the lives of millions of residents.

## Supplementary Material

Supplementary_Material
