# Supplementary material for: Walking for transportation in large Latin American cities: walking-only trips and total walking events and their sociodemographic correlates
Source: Transp Rev. 2021 Aug 14;42(3):296–317. doi: 10.1080/01441647.2021.1966552 (PMC7612619; doi:10.1080/01441647.2021.1966552)
Supplement: Supplementary_Material [file TTRV_A_1966552_SM4294.docx]

| **Table S1.** City characteristics and details of household travel surveys. | | | | |
| --- | --- | --- | --- | --- |
| **City** | **Geographic extent and city characteristics^a^** | **Sample frame** | **Definition of trips and events** | **Survey** |
| Mexico City | “*Zona Metropolitana del Valle de Mexico*” (61 municipalities). Population is 18 million (approx.). Population density is 85 persons/ha. 89% of built-up area is urban. Intersection density is 84 per km^2^. GDP per capita is $21,641. | All population over 5 years old. 200,117 individuals. 1 weekday and 1 weekend day. | The survey is based on trips carried out in one or several means of transportation, with a specific purpose, from a place of origin to another destination. The survey also includes stages, which also consider walking. | [Encuesta Origen Destino en Hogares de la Zona Metropolitana del Valle de México (EOD) 2017](https://www.inegi.org.mx/programas/eod/2017/) |
| Bogota | “*Bogotá y su área de influencia*” (19 municipalities). Population is 8 million (approx.). Population density is 196 persons/ha. 93% of built-up area is urban. Intersection density is 125 per km^2^. GDP per capita is $12,353. | All population over 5 years old. 66,821 individuals. 1 weekday and 1 weekend day. | The survey is based on trips conducted with a specific purpose and that have a duration of more than three minutes. Trips under three minutes are included if the purpose is work or study. Even though the survey includes stages, it does not consider walking as a stage per se. However, stages conducted in transportation modes other than walking include walking time after that stage. Additionally, each trip includes walking time before the trip. | [Encuesta Origen – Destino de Hogares (EODH) para Bogotá y los municipios vecinos de su área de influencia 2019](http://www.simur.gov.co/portal-simur/datos-del-sector/encuestas-de-movilidad/) |
| Santiago de Chile | “*Región Metropolitana de Santiago*” (45 comunas). Population is 6.5 million (approx.). Population density is 85 persons/ha. 89% of built-up area is urban. Intersection density is 123 per km^2^. GDP per capita is $26,563. | All population. 60,054 individuals. 1 day (both considering weekdays and weekends) | The survey is based on trips conducted for a specific purpose, between an origin and a destination, with one or more transportation modes. Even though the survey includes stages, it does not consider walking as a stage per se. However, trips include walking time to reach the destination, and stages include walking time before each stage. | [Encuesta Origen Destino Santiago 2012](http://www.sectra.gob.cl/biblioteca/detalle1.asp?mfn=3253) |
| Sao Paulo | “*Regiaõ Metropolitana de Saõ Paulo*” (39 municipalities). Population is 20 million (approx.). Population density is 93 persons/ha. 92% of built-up area is urban. Intersection density is 80 per km^2^. GDP per capita is $24,921. | All population. 86,319 individuals. 1 weekday. | The survey is based on trips conducted with a specific purpose, between an origin and a destination, using one or more transportation modes. Walking trips are included if the distance travelled is over 500m if the purpose of the trip is not study or work. The database does not include stages. | [Pesquisa Origem e Destino da Região Metropolitana de Saõ Paulo 2017](http://www.metro.sp.gov.br/pesquisa-od/index.aspx) |
| Buenos Aires | “*Área Metropolitana de Buenos Aires*” (44 partidos). Population is 14 million (approx.). Population density is 72 persons/ha. 90% of built-up area is urban. Intersection density is 70 per km^2^. GDP per capita is $28,597. | All population over 3 years old. 70,321 individuals. 1 weekday. | The survey includes trips conducted between an origin and a destination with a specific purpose, and only when the distance travelled is more than two blocks. The survey includes stages, but walking is not included. | [Encuesta de Movilidad Domiciliaria 2009-2010 para el Área Metropolitana de Buenos Aires](http://datar.noip.me/dataset/encuesta-de-movilidad-domiciliaria-2009-2010-amba) |
| a. Data on population (of urban extent), population density and % of urban built-up area was collected from the Atlas of Urban Expansion (<http://www.atlasofurbanexpansion.org/>). Intersection densities were calculated using OSMnx python package, for the urban extent of each city. Data on GDP per capita (purchasing power parity in constant 2011 international USD) was extracted from gridded global datasets for Gross Domestic Product, described in *Kummu, Matti; Taka, Maija; Guillaume, Joseph H. A. (2020), Data from: Gridded global datasets for Gross Domestic Product and Human Development Index over 1990-2015, Dryad, Dataset,* [*https://doi.org/10.5061/dryad.dk1j0*](https://doi.org/10.5061/dryad.dk1j0) | | | | |

| **Table S2.** Sample characteristics of each household survey, unweighted and weighted to population level. | | | | | | | | | | | | | | |  |
| --- | --- | --- | --- | --- | --- | --- | --- | --- | --- | --- | --- | --- | --- | --- | --- |
|  | **Mexico City 2017** | | | **Bogota 2019** | | | **Santiago de Chile 2012** | | | **Sao Paulo 2017** | | | **Buenos Aires 2010** | | |
|  | Sample | Population | %^a^ | Sample | Population | %^a^ | Sample | Population | %^a^ | Sample | Population | %^a^ | Sample | Population | %^a^ |
| **Total** | 188,564 | 19,690,219 | 100.0 | 63,244 | 8,939,564 | 100.0 | 56,326 | 6,190,953 | 100.0 | 82,239 | 19,356,755 | 100.0 | 65,403 | 12,044,417 | 100.0 |
| **Sex** |  |  |  |  |  |  |  |  |  |  |  |  |  |  |  |
| Men | 90,878 | 9,483,275 | 48.2 | 29,686 | 4,189,826 | 46.9 | 26,429 | 3,024,260 | 48.8 | 38,506 | 9,108,011 | 47.1 | 31,823 | 5,720,716 | 47.5 |
| Women | 97,686 | 10,206,944 | 51.8 | 33,558 | 4,749,739 | 53.1 | 29,897 | 3,166,693 | 51.2 | 43,733 | 10,248,745 | 52.9 | 33,580 | 6,323,701 | 52.5 |
| **Age** |  |  |  |  |  |  |  |  |  |  |  |  |  |  |  |
| <18 | 38,645 | 4,013,977 | 20.4 | 11,518 | 1,690,333 | 18.9 | 10,263 | 1,213,854 | 19.6 | 12,642 | 3,566,692 | 18.4 | 14,618 | 2,699,856 | 22.4 |
| 18-29 | 39,344 | 4,097,266 | 20.8 | 14,013 | 1,972,353 | 22.1 | 11,708 | 1,320,574 | 21.3 | 15,051 | 4,050,951 | 20.9 | 12,911 | 2,383,977 | 19.8 |
| 30-64 | 92,094 | 9,649,264 | 49.0 | 29,775 | 4,202,495 | 47.0 | 26,628 | 2,988,630 | 48.3 | 42,531 | 9,926,534 | 51.3 | 28,835 | 5,407,111 | 44.9 |
| 65=< | 18,481 | 1,929,712 | 9.8 | 7,938 | 1,074,383 | 12.0 | 7,727 | 667,896 | 10.8 | 12,015 | 1,812,579 | 9.4 | 9,039 | 1,553,472 | 12.9 |
| **Age-appropriate education level** | | | | |  |  |  |  |  |  |  |  |  |  |  |
| No | 31,530 | 3,213,503 | 16.3 | 12,002 | 1,739,814 | 19.5 | 8,992 | 817,053 | 13.2 | 21,529 | 5,665,249 | 29.3 | 28,928 | 4,967,486 | 41.2 |
| Yes | 156,793 | 16,450,011 | 83.5 | 51,241 | 7,199,619 | 80.5 | 46,807 | 5,320,589 | 85.9 | 60,710 | 13,691,506 | 70.7 | 36,328 | 7,048,241 | 58.5 |
| **SES** |  |  |  |  |  |  |  |  |  |  |  |  |  |  |  |
| Low | 111,343 | 11,096,778 | 56.4 | 28,116 | 4,176,760 | 46.7 | 18,676 | 1,666,400 | 26.9 | 27,161 | 4,176,649 | 21.6 | 18,900 | 3,328,496 | 27.6 |
| Middle | 55,839 | 6,020,872 | 30.6 | 21,934 | 3,104,604 | 34.7 | 18,765 | 1,834,822 | 29.6 | 81,463 | 19,135,181 | 98.9 | 27,447 | 4,871,719 | 40.4 |
| High | 21,382 | 2,572,569 | 13.1 | 13,194 | 1,658,200 | 18.5 | 18,885 | 2,689,730 | 43.4 | 776 | 221,575 | 1.1 | 19,056 | 3,844,202 | 31.9 |
| **Access to car** |  |  |  |  |  |  |  |  |  |  |  |  |  |  |  |
| No | 108,762 | 11,230,815 | 57.0 | 39,244 | 5,789,284 | 64.8 | 32,755 | 3,019,385 | 48.8 | 30,854 | 8,174,326 | 42.2 | 39,276 | 7,173,496 | 59.6 |
| Yes | 79,802 | 8,459,404 | 43.0 | 24,000 | 3,150,281 | 35.2 | 23,571 | 3,171,568 | 51.2 | 51,385 | 11,182,430 | 57.8 | 26,127 | 4,870,921 | 40.4 |
| a. Weighted percentages using survey-specific population weights. | | | | | | | | | | | | | | | |

| **Table S3.** Harmonization of trip purposes across the five household travel surveys. | | | | | | | | | | | | | | | |  |
| --- | --- | --- | --- | --- | --- | --- | --- | --- | --- | --- | --- | --- | --- | --- | --- | --- |
| **Harmonized variable** | **Mexico City 2017** | | | **Bogota 2019** | | | **Santiago de Chile 2012** | | | **Sao Paulo 2017** | | | **Buenos Aires 2010** | | |  |
| Work or study | To work, to study | | | Work, work matters, study | | | To work, for work, to study, for study | | | Work (industry), work (retail), work (services), school/education, job search | | | Workplace, work-related, study, study (other) | | |  |
| Errands | Shopping (goods and services), pick someone up or drop someone off, do an errand, go to the doctor’s office, or receive medical attention | | | Receive medical attention, pick someone up or drop someone off, pick something up or drop something off, errands, take care of someone | | | Health, pick someone up or drop someone off, pick something up or drop something off, shopping, errands | | | Shopping, doctor/dentist/health, personal affairs. We have also considered trips conducted with someone else as ‘pick someone up or drop someone off’ | | | Health, shopping, personal, errands, pick someone up or drop someone off | | |  |
| Recreational destinations | Visits (friends or family), sports or recreation, attend a religious act | | | See someone, eat/drink something, recreation and culture, religious activities, physical activity and sports | | | Visit someone, eating or drinking, recreation | | | Recreation, visits, leisure, meals | | | Sports, recreation, gastronomy, friends, family, social, worship | | |  |
| Other | Other | | | Other | | | Other activities | | | - | | | Other | | |  |
| Return home | Go home | | | Return home | | | Return home | | | Residence | | | Home | | |  |
| Does not know / no answer | Does not know | | | Does not answer | | | - | | | - | | | - | | |  |
| For Mexico City 2017, trip purposes in the survey documentation are: *Ir al trabajo, Ir a estudiar, Ir de compras (bienes y servicios), Llevar o recoger a alguien, Hacer un trámite, Ir al médico o recibir atención de salud, A convivir (amigos o familiares), deportes o recreación, Ir a acto religioso, Otro, Ir al hogar, No sabe*. For Bogota 2019: *Trabajar, Asuntos de trabajo, Estudiar, Recibir atención en salud, Buscar/dejar a alguien, Buscar/dejar algo, Compras, Trámites, Cuidado de personas, Ver a alguien, Comer/tomar algo, Recreación y cultura, Actividades con fines religiosos, Actividad física y deporte, Otro, Volver a casa, No responde*. For Santiago de Chile 2012: *Al trabajo, Por trabajo, Al estudio, Por estudio, De salud, Buscar o Dejar a alguien, Buscar o dejar algo, De compras, Trámites, Visitar a alguien, Comer o Tomar algo, Recreación, Otra actividad, Volver a casa*. For Sao Paulo 2017: *Trabalho Indústria, Trabalho Comércio, Trabalho Serviços, Escola/Educação, Procurar Emprego, Compras, Médico/Dentista/Saúde, Assuntos Pessoais, Recreação/Visitas/Lazer, Refeição, Residência*. For Buenos Aires 2010: *Lugar de trabajo, Asunto laboral, Cursar estudio, Estudios (otro), Salud, compras, Personal, Trámites personales, Dejar, recoger o acompañar a miembros y no miembros del hogar, Deportes, Recreación, Gastronomía, Amigos, Família, Social, Culto, Otro, Hogar*. | | | | | | | | | | | | | | | |  |
| **Table S4.** Harmonization of education level across the five household travel surveys. | | | | | | | | | | | | | | | | |
| **Harmonized variable** | | **Mexico City 2017** | | | **Bogota 2019** | | | | **Santiago de Chile 2012** | | | **Sao Paulo 2017** | | | **Buenos Aires 2010** | |
| *Education level* | | *Last year of school passed* | | | *Maximum education level achieved* | | | | *Studies* | | | *Instruction level* | | | *Education level achieved* | |
| Less than primary | | None (2.4%) + Preschool or kindergarten (2.9%) | | | None (1.3%) + Preschool (1.9%) + Incomplete primary (12.4%) | | | | None or never studied  (0.8%) + Preschool/Nursery school (1.8%) | | | Not literate/ Incomplete fundamental I (15.3%) | | | None  (2.5%) + Incomplete primary/EGB (17.6%) + Informal education (0.1%) | |
| Primary | | Primary (23.4%) | | | Primary (7.4%) + Incomplete secondary (11.9%) | | | | Basic/Primary (24.7%) | | | Fundamental I/Incomplete fundamental II  (16%) + Fundamental II/Incomplete middle (15.7%) | | | Primary/EGB (20%) +  Incomplete secondary/multimodal  (20.3%) | |
| Secondary | | Secondary (25.4%) + Preparatory or baccalaureate (20.8%) | | | Secondary (6.6%) + Incomplete middle (10^th^-11th) (3.5%) + Middle (10^th^-11th) (16.6%) + Incomplete technical /technological (2.3%) + Incomplete university degree (7.1%) | | | | Middle scientific/humanist (31.4%) + Humanities (3.9%) + Middle technical-professional (8.2%) | | | Middle/Incomplete superior (37.3%) | | | Secondary/multimodal  (18.6%) + Incomplete tertiary (3.0%) + Incomplete university (6%) | |
| Superior | | Technical degree with finished secondary  (2%) + Technical degree with finished preparatory  (2.8%) + Normal basic (0.2%) + Bachelor’s degree or professional degree  (18.3%) + Master’s degree or PhD (1.5%) | | | Technical/technological (10.4%) + University degree (12.8%) + Incomplete postgraduate (0.5%) + Postgraduate (5.2%) | | | | Technical training centre (2.5%) + Normalist (0.1%) + Professional institute (5.5.%) + University degree (20%) | | | Superior (15.7%) | | | Tertiary (5.4%) + University (5.5%) + Postgraduate studies (0.7%) | |
| For Mexico City 2017, education levels in the survey documentation are: *Ninguno, Preescolar o kinder, Primaria, Secundaria, Preparatoria o bachillerato, Normal básica, Carrera técnica con secundaria terminada, Carrera técnica con preparatoria terminada, Licenciatura o professional, Maestría o doctorado*. For Bogota 2019: *Ninguno, Preescolar, Primaria incompleta, Primaria completa, Secundaria incompleta, Secundaria completa, Media incompleta, Media completa, Técnico/Tecnológico incompleto, Universitario incompleto, Técnico/Tecnológica completa, Universitario completo, Postgrado incompleto, Postgrado completo*. For Santiago de Chile 2012: *Ninguno o nunca estudió, Preescolar/Parvularia, Básica/Primaria, Media Científica/humanista, Humanidades, Media Técnico-Profesional, Normalista, Centro de Formación Técnica, Instituto Profesional, Universitaria*. For Sao Paulo 2017: *Não Alfabetizado/ Fundamental I Incompleto, Fundamental I Completo/ Fundamental II Incompleto, Fundamental II Completo/ Médio Incompleto, Médio Completo/ Superior Incompleto, Superior Completo*. For Buenos Aires 2010: *Sin estudios, Primario incompleto/EGB incompleto, Educación no formal, Primario completo/EGB completo, Secundario incompleto/Polimodal incompleto, Secundario completo/Polimodal completo, Terciario incompleto, Universitario incompleto, Terciario completo, Universitario completo, Estudios de postgrado.* | | | | | | | | | | | | | | | | |
| **Table S5.** Harmonization of socioeconomic level across the five household travel surveys. | | | | | | | | | | | | | |  | | |
| **Harmonized variable** | | | **Mexico City 2017** | | | **Bogota 2019** | | **Santiago de Chile 2012** | | | **Sao Paulo 2017** | | | **Buenos Aires 2010** | | |
| *Socioeconomic Level (SES)* | | | *Household socioeconomic status* | | | *Household income groups* | | *Tertiles of household monthly income (Chilean Pesos)* | | | *Tertiles of household monthly income (Reais)* | | | *Strata based on household income* | | |
| Low | | | Low (0.9%) + Medium-low (58.6%) | | | 1 (23.5%) + 2 (21.2%) | | <= 420,000 (33.4%) | | | <= 2,700 (33.4%) | | | 1 (28.7%) | | |
| Middle | | | Medium-high (29.3%) | | | 3 (11.5%) + 4 (8.2%) + 5 (8.2%) + 6 (6.5%) | | 420,201-780,000 (33.4%) | | | 2,701-4,898 (32.6%) | | | 2 (21.6%) + 3 (18.6%) | | |
| High | | | High (11.2%) | | | 7 (5.0%) + 8 (3.1%) + 9 (3.5%) + 10 (9.1%) | | 780,001 =< (33.2%) | | | 4,899 =< (33.0%) | | | 4 (16.5%) + 5 (14.6%) | | |
| For Mexico City 2017, “Household socioeconomic status” refers to “ESTRATO” in the survey, defined as “*Estrato sociodemográfico*”, and can take the following values: “*Bajo*”, “*Medio bajo*”, “*Medio alto*” and “*Alto”*. For Bogotá 2019, “Household income groups” refers to “id_rango_ingresos” in the survey, defined as “*Ingresos por hogar*”, and can take values from 1 to 10 (from low to high income). For Santiago de Chile 2012, “Tertiles of household monthly income” refers to “IngresoHogar” in the survey, calculated in Chilean Pesos. For Sao Paulo 2017, “Tertiles of household monthly income” refers to “*Renda familiar mensal*” in the survey, calculated in Brazilian Reais. For Buenos Aires 2010, “Strata based on household income” refers to “Quintil_ing_per_capita_AD_EQUIV”, defined as “*QUINTIL ingreso per capita por adulto equivalente del hogar*”, and can take values from 1 to 5. | | | | | | | | | | | | | | | | |

| **Table S6.** P-values of coefficients from regression models for individual walking-only trip indicators stratified by city, related to the estimated values presented in Table 2. | | | | | | | | | | | | | | | | | | | | |
| --- | --- | --- | --- | --- | --- | --- | --- | --- | --- | --- | --- | --- | --- | --- | --- | --- | --- | --- | --- | --- |
|  | **Daily walking-only trips per capita (WOT)** | | | | | **% of all trips that are walking-only (%WOT)** | | | | | **Daily time spent in walking-only trips (TWOT)** | | | | | **% of population meeting WHO PA guidelines from walking-only trips on weekdays (%GT)** | | | | |
|  | MX17 | BO19 | SC12 | SP17 | BA10 | MX17 | BO19 | SC12 | SP17 | BA10 | MX17 | BO19 | SC12 | SP17 | BA10 | MX17 | BO19 | SC12 | SP17 | BA10 |
| **Sex (Ref.=Men)** | |  |  |  |  |  |  |  |  |  |  |  |  |  |  |  |  |  |  |  |
| Women |  |  |  |  |  |  |  |  |  |  |  |  |  |  |  |  |  |  |  |  |
| **Age (Ref.=<18)** |  |  |  |  |  |  |  |  |  |  |  |  |  |  |  |  |  |  |  |  |
| 18-29 |  |  |  |  |  |  |  |  |  |  |  |  |  |  |  |  |  |  |  |  |
| 30-64 |  |  |  |  |  |  |  |  |  |  |  |  |  |  |  |  |  |  |  |  |
| 65=< |  |  |  |  |  |  |  |  |  |  |  |  |  |  |  |  |  |  | 0.178 |  |
| **Age-appropriate education level (Ref.=No)** | | | | | |  |  |  |  |  |  |  |  |  |  |  |  |  |  |  |
| Yes |  | 0.144 | 0.263 |  |  |  |  |  |  |  |  |  | 0.506 |  |  |  | 0.028 | 0.400 |  |  |
| **SES (Ref.=Low)** |  |  |  |  |  |  |  |  |  |  |  |  |  |  |  |  |  |  |  |  |
| Middle |  |  |  |  |  |  |  |  |  |  |  |  |  |  |  |  |  | 0.106 |  |  |
| High |  |  |  |  |  |  |  |  |  |  |  |  |  |  |  |  |  | 0.028 |  |  |
| **Car ownership (Ref.=No)** | | | | |  |  |  |  |  |  |  |  |  |  |  |  |  |  |  |  |
| Yes |  |  | 0.01 |  |  |  |  |  |  |  |  |  |  |  |  |  |  |  |  |  |
| P-values of coefficients are <0.01 except where shown. | | | | | | | | | | | | | | | | | | | | |

| **Table S7.** P-values of coefficients from regression models for walking-only trip purposes stratified by city, related to the estimated values presented in Table 3. | | | | | | | | | | | | | | | |  |
| --- | --- | --- | --- | --- | --- | --- | --- | --- | --- | --- | --- | --- | --- | --- | --- | --- |
|  | **% Work or study** | | | | | **% Errands** | | | | | **% Recreational destinations** | | | | | |
|  | MX17 | BO19 | SC12 | SP17 | BA10 | MX17 | BO19 | SC12 | SP17 | BA10 | MX17 | BO19 | SC12 | SP17 | BA10 | |
| **Sex (Ref.=Men)** | |  |  |  |  |  |  |  |  |  |  |  |  |  |  | |
| Women |  |  |  |  |  |  |  |  |  |  |  |  |  |  |  | |
| **Age (Ref.=<18)** |  |  |  |  |  |  |  |  |  |  |  |  |  |  |  | |
| 18-29 |  |  |  |  |  |  |  |  |  |  |  |  |  |  |  | |
| 30-64 |  |  |  |  |  |  |  |  |  |  |  |  | 0.172 |  |  | |
| 65=< |  |  |  |  |  |  |  |  |  |  |  |  | 0.064 |  |  | |
| **Age-appropriate education level (Ref.=No)** | | | |  |  |  |  |  |  |  |  |  |  |  |  | |
| Yes | 0.300 | 0.103 | 0.015 |  |  | 0.991 | 0.909 | 0.754 | 0.340 | 0.351 | 0.677 | 0.750 |  |  | 0.960 | |
| **SES (Ref.=Low)** |  |  |  |  |  |  |  |  |  |  |  |  |  |  |  | |
| Middle | 0.210 | 0.979 |  |  | 0.051 |  | 0.044 | 0.056 |  |  |  |  | 0.430 |  |  | |
| High | 0.435 | 0.840 |  |  |  |  |  |  |  |  |  |  | 0.476 |  |  | |
| **Car ownership (Ref.=No)** | |  |  |  |  |  |  |  |  |  |  |  |  |  |  | |
| Yes |  | 0.032 | 0.510 |  |  |  | 0.280 | 0.327 |  | 0.498 |  | 0.013 |  |  | 0.247 | |
| P-values of coefficients are <0.01 except where shown. | | | | | | | | | | | | | | | | |

| **Table S8.** P-values of coefficients from regression models for individual walking event indicators stratified by city, related to the estimated values presented in Table 4. | | | | | | | | | | | | | | | |
| --- | --- | --- | --- | --- | --- | --- | --- | --- | --- | --- | --- | --- | --- | --- | --- |
|  | **Daily total walking events per capita (TWE)** | | | **% of all events that are walking (%TWE)** | | | **% of total walking events that are part of a multimodal trip (%M)** | | | **Daily time spent in total walking events (TTWE)** | | | **% of population meeting WHO PA guidelines from total walking events on weekdays (%GE)** | | |
|  | MX17 | BO19 | SC12 | MX17 | BO19 | SC12 | MX17 | BO19 | SC12 | MX17 | BO19 | SC12 | MX17 | BO19 | SC12 |
| **Sex (Ref.=Men)** | |  |  |  |  |  |  |  |  |  |  |  |  |  |  |
| Women |  |  |  |  |  |  |  |  |  |  |  |  |  |  |  |
| **Age (Ref.=<18)** |  |  |  |  |  |  |  |  |  |  |  |  |  |  |  |
| 18-29 |  |  |  |  |  | 0.503 |  |  |  |  | 0.106 |  |  |  |  |
| 30-64 | 0.123 |  |  |  |  |  |  |  |  |  | 0.189 |  |  |  |  |
| 65=< |  |  | 0.042 |  |  | 0.215 |  |  |  |  | 0.441 | 0.712 |  |  |  |
| **Age-appropriate education level (Ref.=No)** | | | | | |  |  |  |  |  |  |  |  |  |  |
| Yes |  |  | 0.085 |  |  |  |  |  |  | 0.828 | 0.995 | 0.154 | 0.027 |  | 0.394 |
| **SES (Ref.=Low)** |  |  |  |  |  |  |  |  |  |  |  |  |  |  |  |
| Middle |  |  | 0.821 |  | 0.020 |  |  |  |  |  |  | 0.082 |  |  | 0.808 |
| High |  |  | 0.023 |  |  |  |  |  |  |  |  | 0.668 |  |  | 0.262 |
| **Car ownership (Ref.=No)** | | |  |  |  |  |  |  |  |  |  |  |  |  |  |
| Yes |  |  |  |  |  |  | 0.128 |  |  |  |  |  |  |  |  |
| P-values of coefficients are <0.01 except where shown. | | | | | | | | | | | | | | | |
